# Clinical application of different administration methods of tranexamic acid in transforaminal lumbar interbody fusion surgery

**DOI:** 10.3389/fmed.2026.1777031

**Published:** 2026-03-30

**Authors:** Wei Ye, Jianshu Shao, Hanke Zhao

**Affiliations:** 1 Department of Orthopedic Surgery, Wujin Hospital Affiliated with Jiangsu University, Changzhou, China; 2 Department of Orthopedic Surgery, The Wujin Clinical College of Xuzhou Medical University, Changzhou, China

**Keywords:** hemorrhage, intravenous injection, oral, tranexamic acid, transforaminal lumbar interbody fusion

## Abstract

**Aim:**

The purpose of this research was to investigate the effectiveness and safety associated with various administration routes of tranexamic acid (TXA) in transforaminal lumbar interbody fusion (TLIF) procedures.

**Methods:**

A cohort of 60 patients who underwent standard TLIF procedures was enrolled and categorized into three groups based on distinct TXA administration strategies: Group A (received preoperative intravenous TXA injection), Group B (received preoperative intravenous TXA injection followed by postoperative oral TXA for 3 days), and Group C (received preoperative intravenous TXA injection followed by postoperative oral TXA for 5 days). Comparative analyses were conducted among the three groups concerning surgical blood loss, operation time, length of hospital stay, hemoglobin and hematocrit levels, coagulation and fibrinolytic markers, transfusion requirements and transfusion rates, incidence of complications, and lumbar spine functionality.

**Results:**

In comparison to Group A, Groups B and C exhibited reduced total blood loss, concealed blood loss, and postoperative drainage volumes, along with shorter hospital stays. Additionally, Groups B and C demonstrated higher hemoglobin and hematocrit levels, along with lower transfusion rates (*p* < 0.05). Notably, no significant disparities were seen between Groups B and C across these observed metrics (*p* > 0.05). Furthermore, no substantial differences were identified among the three groups in terms of intraoperative blood loss, drainage tube removal time, coagulation and fibrinolytic markers, complication rates, and JOA and ODI scores one-year post-operation (*p* > 0.05).

**Conclusion:**

Administration protocol involving preoperative intravenous TXA injection followed by 3 days of postoperative oral TXA can effectively decrease total and concealed blood loss, reduce postoperative drainage, shorten hospital stays, elevate hemoglobin and hematocrit levels, and diminish transfusion rates in patients undergoing TLIF surgery. This combined regimen shows a safety profile comparable to preoperative intravenous TXA alone, with no increase in complications or adverse events. Extending oral TXA to 5 days does not provide additional benefit.

## Introduction

Recently, owing to the escalating aging of the population, coupled with the prevalence of unhealthy lifestyles and living patterns, the incidence of degenerative lumbar spinal stenosis (DLSS) has gradually increased. Globally, 3.6% of the population is affected by DLSS (1). There is still a lack of large-scale epidemiological investigations in China, but some articles have pointed out that the prevalence of DLSS in China is 3.9–11.0%, and this rate is also increasing (2). Consequently, DLSS has become one of the health problems affecting people’s quality of life.

Transforaminal lumbar interbody fusion (TLIF) belongs to the main surgical method for treating DLSS, which relieves the compression of nerve roots and dural sac through spinal canal decompression (3). The main advantages of TLIF include mature technology, sufficient surgical field, significant therapeutic effect, thorough decompression, and high intervertebral bone graft fusion rate (4). At the same time, excessive intraoperative bleeding is a common complication of TLIF (5). Damage to the nutrient vessels of the lumbar and dorsal muscles, bleeding from the bone surface of the vertebrae, and bleeding from the venous plexus within the spinal canal can lead to an increase in intraoperative blood loss along with postoperative drainage volume, resulting in unstable postoperative hemodynamics and an increased probability of postoperative complications (6). Therefore, how to control blood loss during TLIF surgery has become a concern for spinal surgeons.

Tranexamic acid (TXA) has been widely investigated in spinal surgery over the past decade. A growing body of evidence from randomized controlled trials and meta-analyses has demonstrated that TXA effectively reduces perioperative blood loss and transfusion requirements in various spinal procedures (7–9). However, the optimal administration strategy for TXA in TLIF surgery remains unclear.

Previous studies have primarily focused on single administration routes, such as intravenous bolus, continuous infusion, or topical application (10). However, the fibrinolytic response to surgical trauma peaks at 24–72 h postoperatively, suggesting that a single preoperative dose may not provide adequate coverage during this critical period (11). The concept of combined intravenous and oral TXA administration has emerged as a potential strategy to maintain therapeutic drug levels throughout the postoperative fibrinolytic window. While this approach has been successfully implemented in hip and knee arthroplasty (12), its efficacy and safety in TLIF surgery have not been thoroughly evaluated.

Therefore, we planned to compare the clinical effects of different administration methods of TXA in TLIF surgery. The selection of 3-day and 5-day postoperative oral durations was based on both pharmacological and clinical evidence: (1) TXA has a plasma half-life of approximately 2 h, and sustained oral administration can maintain stable anti-fibrinolytic activity during the peak fibrinolytic period (24–72 h postoperatively), which is critical for reducing delayed and hidden blood loss; (2) Previous studies in joint replacement surgeries have demonstrated that 3–5 days of oral TXA effectively supplements intravenous administration, while longer durations do not improve efficacy (13). This study aimed to explore the optimal administration route and guide the rational application of TXA in TLIF surgery in future clinical work.

## Materials and methods

### Study design

This was a prospective observational cohort study conducted at our institution between January 2020 and December 2022. A total of 60 patients who underwent conventional TLIF surgery were consecutively enrolled and assigned to three groups based on clinical TXA administration strategies according to surgeon preference and patient informed consent (non-randomized): Group A (Preoperative intravenous injection of TXA), Group B (Preoperative intravenous injection of TXA + postoperative oral administration of TXA for 3 days), and Group C (Preoperative intravenous injection of TXA + postoperative oral administration of TXA for 5 days), with 20 cases in each group. A CONSORT flow diagram detailing patient enrollment, inclusion/exclusion, and group allocation is provided in Supplementary Figure S1.

*Inclusion criteria*: (1) Diagnosed with DLSS-related diseases such as lumbar intervertebral disc protrusion, lumbar spinal canal stenosis, and lumbar spondylolisthesis; and kidney functions were all normal; (2) The patient underwent the first conventional transforaminal approach lumbar interbody fusion surgery. (3) Those who were not allergic to TXA drugs; (4) Preoperative blood routine, coagulation function as well as liver and kidney functions were all normal; (5) There was no history of thrombosis before the operation, and the preoperative color Doppler ultrasound report of the lower extremity veins showed no thrombosis and no thrombosis in the intermuscular veins.

*Exclusion criteria*: (1) The patient has a history of lumbar surgery or other spinal disorders; (2) Patients with postoperative cerebrospinal fluid leakage; (3) Patients with a history of hemophilia, moderate to severe cardiac dysfunction, cerebral hemorrhage and cerebral infarction; (4) The patient took drugs to prevent platelet aggregation or antithrombotic drugs before the operation.

### Sample size calculation

A pre-specified power analysis was performed using G*Power 3.1 software to determine the sample size. For the primary endpoint (total blood loss) as described previously (14), with *α* = 0.05 (two-tailed) and power (1 − *β*) = 0.80, the calculated minimum sample size per group was 18. We enrolled 20 patients per group to account for potential dropouts.

### Surgery methods

The patient was administered general anesthesia, underwent standard disinfection procedures, and was then positioned prone with the focus centered on the level of the adjacent vertebral intervertebral disc. A median longitudinal skin incision was made, approximately 8–10 cm long, to expose the spinous processes, upper and lower facet joints, lamina, and transverse processes of the affected segment. The midpoint where the midline intersected the outer edge of the facet joint was selected as the needle insertion point. Positioning pins were placed on both sides, and after confirmation by fluoroscopy, the pedicle screws were inserted. A bone knife was used to cut the lower facet process on the affected segment, then the medial and upper edge of the upper facet process were severed, and the intervertebral foramen was opened. After decompression, the yellow ligament was severed to expose the dura mater, the nerve root canal was expanded, and the nerve roots were fully exposed and released. The intervertebral disc was removed and the cartilage endplate was treated. The intervertebral space was rinsed, and an appropriate intervertebral fusion device was placed. The internal fixation position was confirmed by fluoroscopy. Complete hemostasis was achieved and the incision was rinsed. A drainage tube was carefully inserted at the site of the surgical incision, and the incision was subsequently sutured in a meticulous, layer-by-layer manner.

### Administration methods of TXA

#### Group A

Thirty minutes prior to the surgical procedure, an intravenous dose of TXA (manufactured by Brilliant Pharmaceutical Co. Ltd.) at 10 mg/kg was administered, and this dosage was sustained at a rate of 1 mg/kg per hour throughout the operation.

#### Group B

The intravenous administration of TXA was the same as Group A, and TXA tablets (China Resources Double-Crane Pharmaceutical Co. Ltd.) were orally administered continuously for 3 days after the surgery, 3 times a day, 1 g each time.

#### Group C

The intravenous administration of TXA was the same as Group A, and TXA tablets were orally administered continuously for 5 days after the surgery, 3 times a day, 1 g each time.

### Postoperative management

All patients received routine cephalosporin antibiotics for 24–48 h to prevent infection.

Postoperative antithrombotic therapy: Early ambulation and intermittent pneumatic compression were applied for venous thromboembolism prophylaxis. Chemical anticoagulation (low-molecular-weight heparin) was administered in patients with high thromboembolic risk according to individual evaluation, starting 12–24 h postoperatively.

Patients were encouraged to perform leg-raising exercises. Blood routine was tested at 96 h postoperatively. Lumbar X-ray and lower extremity venous Doppler ultrasound were performed on postoperative day 5.

The drainage tube was removed when 24-h drainage volume was <50 mL.

Blood transfusion was indicated when hemoglobin <70 g/L, or <80 g/L with symptomatic anemia.

Discharge criteria: (1) Drainage <50 mL/24 h; (2) Afebrile for 24 h; (3) Stable hemodynamics; (4) Able to walk with lumbar brace; (5) No severe complications.

### Observation indicators

(1) The total blood loss volume, hidden blood loss, intraoperative blood loss, postoperative drainage volume, and drainage tube removal time among the three groups were compared. The total blood loss can be calculated using the following formula: Total blood loss = Preoperative patient blood volume × [(Preoperative hematocrit (Hct) − Lowest postoperative Hct)/(Average of preoperative and postoperative Hct)], where the preoperative blood volume is estimated according to the NADLER method. Hidden blood loss is determined by the formula: Hidden blood loss = Total blood loss − Intraoperative blood loss − Postoperative drainage volume.
(2) The operation time and hospital stay among the three groups were compared.
(3) A total of 5 mL of venous blood samples were drawn from the patient prior to surgery, as well as on the 1st, 3rd, and 5th days following the operation. These samples were then analyzed to measure the levels of hemoglobin, hematocrit, prothrombin time (PT), activated partial thromboplastin time (APTT), D-dimer, and fibrinogen (FIB).
(4) The number of blood transfusions and the blood transfusion rate of patients were recorded and calculated.
(5) From the day of the patient’s surgery until 30 days after the operation, during which time the condition of the surgical incision was closely monitored, any abnormal phenomena were promptly recorded. Once the patient met the discharge criteria, they were discharged. Within the 30-day period after the surgery, through telephone follow-ups or patient visits to the clinic, the number of patients with deep venous thrombosis, pulmonary embolism, and incision infection was counted.
(6) The lumbar spine function of the patients was assessed both before surgery and 1 year postoperatively, utilizing the Japanese Orthopedic Association (JOA) Score and the Oswestry Disability Index (ODI). The JOA score spans from 0 to 29, where a higher score signifies superior lumbar spine function. Conversely, the ODI score ranges from 0 to 100, with a higher score indicating more pronounced functional impairment.

### Statistical analysis

The statistical analysis was carried out utilizing SPSS 23.0 software. Normally distributed data were presented as (mean ± standard deviation, *x* ± *s*). For repeated measure measured at different time pointe, repeated-measures ANOVA was used to assess main effects of group, time, and group × time interaction. Post-hoc pairwise comparisons were performed using Bonferroni correction. For non-repeated continuous outcomes, one-way ANOVA was used, followed by LSD-*t* tests. Categorical data were described using numbers and percentages, and Fisher’s exact test was applied for their comparison. There were no missing data for any of the variables analyzed in this study (including demographic characteristics, surgical outcomes, laboratory parameters, and functional scores). Complete data were available for all 60 patients across all time points. Therefore, no imputation methods were required, and all analyses were performed on the full dataset. To address confounding, multivariable linear regression was used for continuous outcomes and multivariable logistic regression for binary outcomes. Adjusted *β* (linear) or odds ratios (OR, logistic) with 95% confidence intervals (CI) are reported. A *p*-value <0.05 was considered statistically significant.

## Results

### General data among the three groups

No significant differences were seen in the general data (gender, age, course of disease, BMI, history of hypertension and history of diabetes) among the three groups (*p* > 0.05, Table 1).

**Table 1:** General data among the three groups.

Items	Group A (*n* = 20)	Group B (*n* = 20)	Group C (*n* = 20)	*p*-value
Gender				0.946
Male	12 (60.00)	10 (50.00)	11 (55.00)	
Female	8 (40.00)	10 (50.00)	9 (45.00)	
Age (years)	56.08 ± 5.06	56.12 ± 5.10	56.18 ± 5.12	
Course of disease (years)	7.52 ± 2.16	7.58 ± 2.23	7.46 ± 2.12	0.998
BMI (kg/m^2^)	24.93 ± 3.20	25.12 ± 3.25	25.06 ± 3.21	0.982
Hypertension				0.944
Yes	8 (40.00)	7 (35.00)	9 (45.00)	
No	12 (60.00)	13 (65.00)	11 (55.00)	
Diabetes				0.929
Yes	5 (25.00)	6 (30.00)	4 (20.00)	
No	15 (75.00)	14 (70.00)	16 (80.00)	

### Surgical blood loss, operation time and hospital stay among the three groups

Surgical blood loss, operation time, and hospital stay among the three groups were analyzed. When compared to Group A, both Group B and Group C exhibited significantly lower volumes of total blood loss (Group A: 1035 ± 107 mL; Group B: 863 ± 86 mL; Group C: 865 ± 87 mL; *p* < 0.001), hidden blood loss (Group A: 400 ± 40 mL; Group B: 320 ± 30 mL; Group C: 318 ± 32 mL; *p* < 0.001), and postoperative drainage (Group A: 320 ± 33 mL; Group B: 220 ± 22 mL; Group C: 218 ± 20 mL; *p* < 0.001), as well as shorter hospital stay (Group A: 7.4 ± 2.3 days; Group B: 5.8 ± 1.1 days; *p* = 0.007; Group C: 5.1 ± 0.7 days; *p* < 0.001). However, no statistically significant differences were observed in total blood loss, hidden blood loss, postoperative drainage volume, or hospital stay between Group B and Group C (*p* > 0.05 for all). Regarding intraoperative blood loss, the time required for drainage tube removal and operation time, there were no notable differences among the three groups (*p* > 0.05 for all).

**Figure 1:**
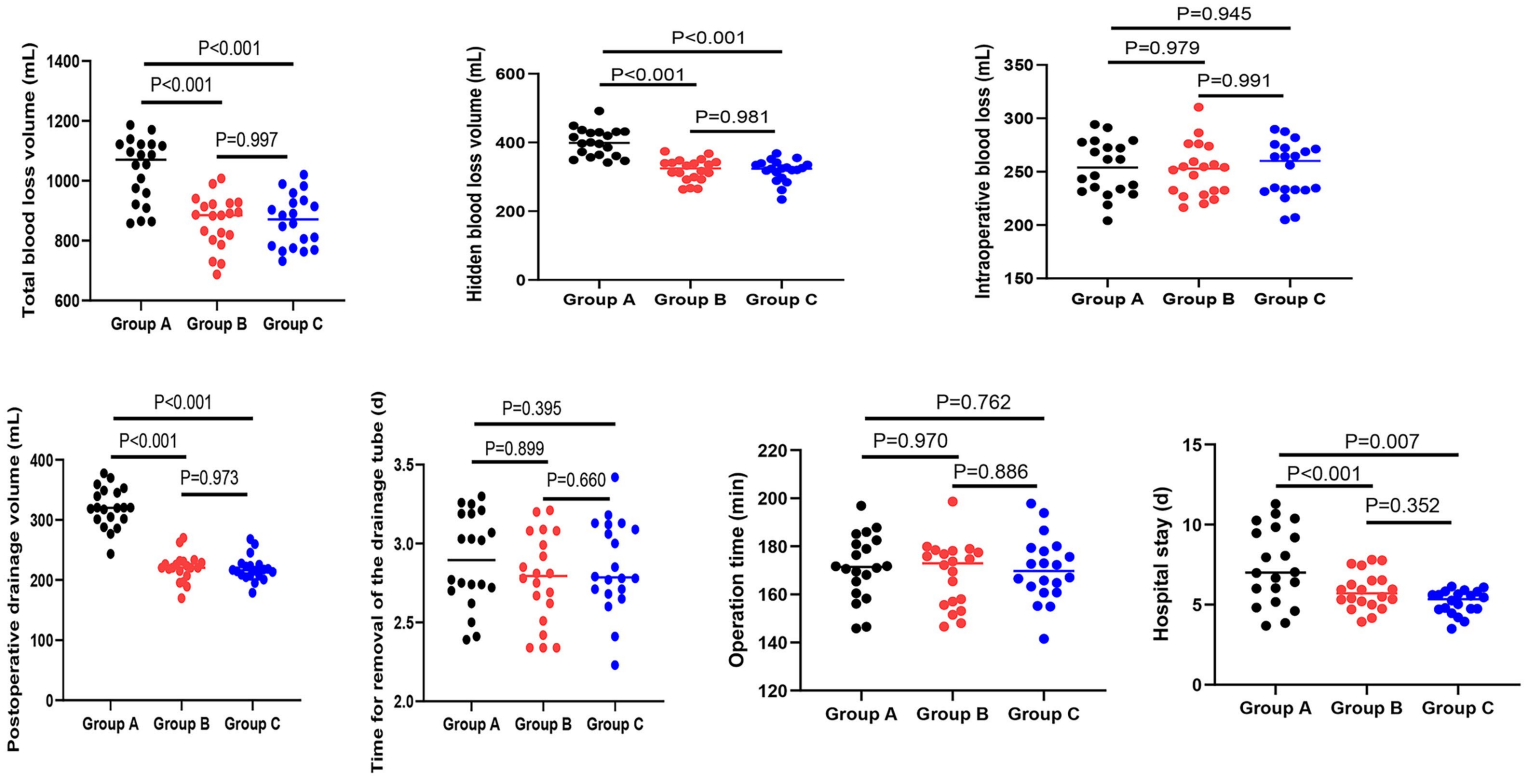
Surgical blood loss, operation time, and hospital stay among the three groups. Data are shown as mean ± SD. Sample sizes: Group A (*n* = 20), Group B (*n* = 20), Group C (*n* = 20).

To address potential confounding, multivariable linear regression analyses were performed adjusting for age, BMI, history of hypertension, and history of diabetes. After adjustment, group allocation remained an independent predictor for all primary outcomes. Compared to Group A, Group B and Group C were both independently associated with significantly lower total blood loss (Group B: *β* = −172.4, 95% CI: −228.6 to −116.2, *p* < 0.001; Group C: *β* = −170.2, 95% CI: −226.4 to −114.0, *p* < 0.001), hidden blood loss (Group B: *β* = −80.3, 95% CI: −98.5 to −62.1, *p* < 0.001; Group C: *β* = −82.1, 95% CI: −100.3 to −63.9, *p* < 0.001), postoperative drainage (Group B: *β* = −100.5, 95% CI: −118.2 to −82.8, *p* < 0.001; Group C: *β* = −102.3, 95% CI: −120.0 to −84.6, *p* < 0.001), and shorter hospital stay (Group B: *β* = −1.6, 95% CI: −2.5 to −0.7, *p* = 0.001; Group C: *β* = −2.3, 95% CI: −3.2 to −1.4, *p* < 0.001). Consistent with the univariate analysis, no significant differences were observed between Group B and Group C after multivariable adjustment for any of these outcomes (*p* > 0.05 for all). These findings confirm that the observed benefits of Groups B and C over A are independent of the measured confounders (see Figure 1).

### Levels of hemoglobin and hematocrit, coagulation function indicators, and fibrinolytic indicators among the three groups

**Figure 2:**
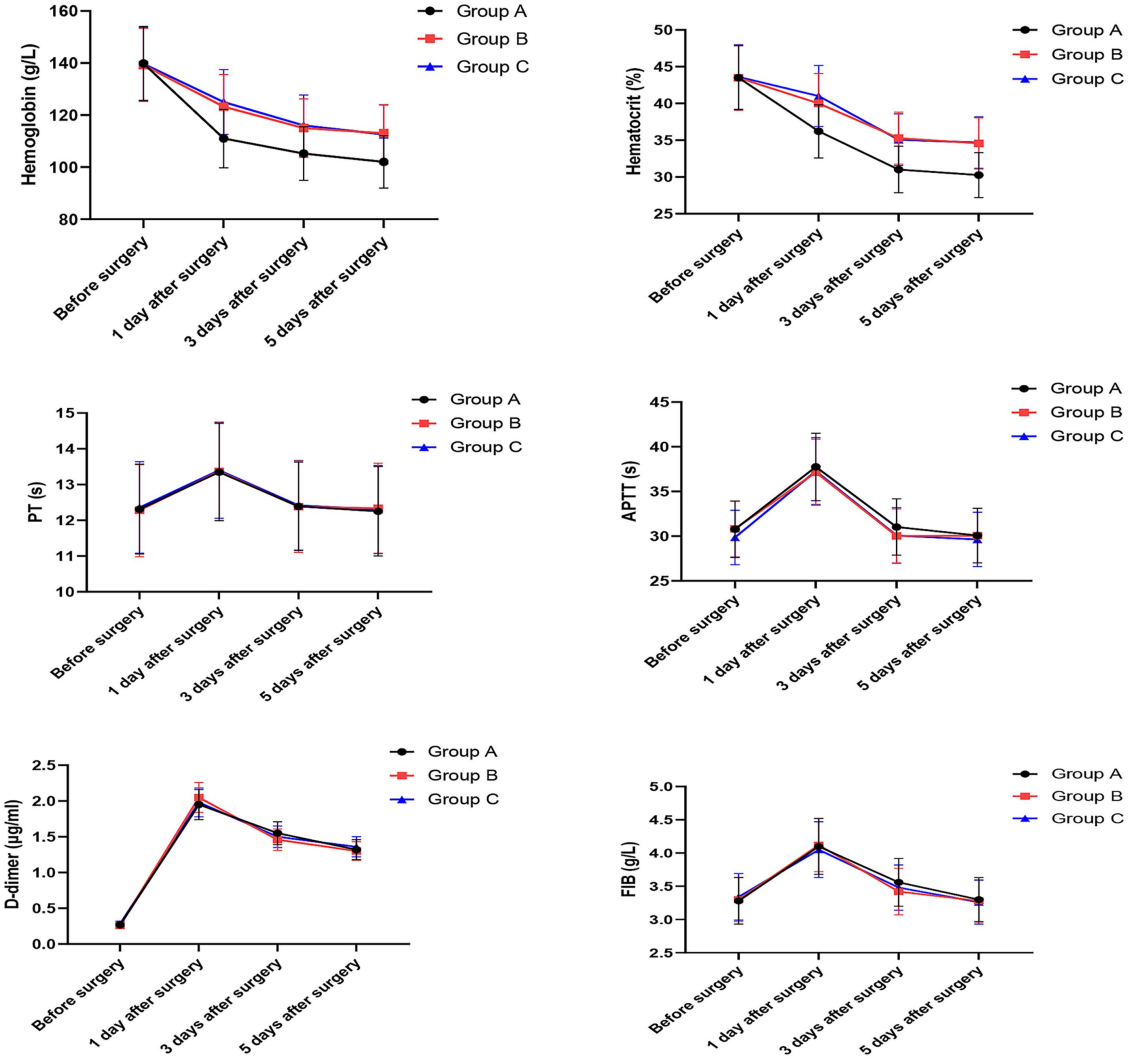
Levels of hemoglobin, hematocrit, coagulation function indicators, and fibrinolytic indicators among the three groups. Data are shown as mean ± SD. Sample sizes: Group A (*n* = 20), Group B (*n* = 20), Group C (*n* = 20).

As shown in Figure 2, there were no significant differences in the levels of hemoglobin and hematocrit among the three groups before surgery (Hb: Group A: 139.8 ± 14.23 g/L, Group B: 139.2 ± 14.0 g/L, Group C: 139.6 ± 13.9 g/L, *p* > 0.05; Hct: Group A: 43.5 ± 4.3%, Group B: 43.4 ± 4.4%, Group C: 43.6 ± 4.4%, *p* > 0.05). Compared with before surgery, the levels of hemoglobin and hematocrit in all three groups were significantly decreased at POD1, POD3 and POD5 (*p* < 0.05 for all).

Two-way repeated measures ANOVA revealed a significant group × time interaction for both hemoglobin (*F* = 4.28, *p* = 0.001) and hematocrit (*F* = 4.15, *p* = 0.002), indicating that the patterns of change over time differed significantly among the three groups.

To adjust for potential confounders, linear mixed models were performed. After adjusting for age, BMI, history of hypertension, and history of diabetes, the group × time interaction remained significant for both hemoglobin (*F* = 4.12, *p* = 0.002) and hematocrit (*F* = 4.01, *p* = 0.003), confirming that the observed differences in trajectories were independent of these covariates. Estimated marginal means from the adjusted models confirmed the unadjusted findings:

At POD1, the hemoglobin levels in Groups B and C were significantly higher than in Group A (Group A: 111.0 ± 11.2 g/L; Group B: 123.2 ± 12.3 g/L; Group C: 125.0 ± 12.4 g/L; *p* = 0.004 and *p* < 0.001), and this difference persisted at POD3 (Group A: 105.2 ± 10.2 g/L; Group B: 115.0 ± 11.2 g/L; Group C: 116.0 ± 11.6 g/L; *p* = 0.029 and *p* = 0.014) and POD5 (Group A: 102.0 ± 10.0 g/L; Group B: 113.0 ± 11.0 g/L; Group C: 112.5 ± 11.3 g/L; *p* = 0.012 and *p* = 0.017). Similar trends were observed for hematocrit levels at POD1 (Group A: 36.2 ± 3.6%; Group B: 40.0 ± 4.0%; Group C: 41.0 ± 4.1%; *p* = 0.004 and *p* < 0.001), POD3 (Group A: 31.0 ± 3.1%; Group B: 35.2 ± 3.5%; Group C: 35.0 ± 3.5%; *p* = 0.001 and *p* = 0.002), and POD5 (Group A: 30.2 ± 3.0%; Group B: 34.5 ± 3.4%; Group C: 34.6 ± 3.5%; *p* = 0.001 and *p* < 0.001).

However, no significant differences were observed between Group B and Group C at any time point for either hemoglobin or hematocrit (*p* > 0.05 for all).

Regarding coagulation function, there were no significant differences in the levels of PT, APTT, D-dimer, and FIB among the three groups at any time point (*p* > 0.05 for all). Two-way repeated measures ANOVA showed no significant group × time interaction for any coagulation parameter (PT: *F* = 0.56, *p* = 0.812; APTT: *F* = 0.48, *p* = 0.876; D-dimer: *F* = 0.72, *p* = 0.678; FIB: *F* = 0.63, *p* = 0.754), indicating that the temporal patterns of these parameters were similar across all three groups. Consistent with these findings, linear mixed models adjusting for age, BMI, history of hypertension, and history of diabetes confirmed the absence of significant group × time interactions for all coagulation parameters (PT: *F* = 0.52, *p* = 0.834; APTT: *F* = 0.44, *p* = 0.892; D-dimer: *F* = 0.68, *p* = 0.702; FIB: *F* = 0.58, *p* = 0.786), demonstrating that the null findings were robust to adjustment for confounders.

### Number of blood transfusions and the blood transfusion rate of patients among the three groups

In Group A, 8 patients (40.00%, 8/20) received blood transfusions; in Group B, 1 patient (5.00%, 1/20) received a transfusion; and in Group C, 1 patient (5.00%, 1/20) received a transfusion. Univariate analysis showed that Groups B and C had significantly lower transfusion rates than Group A (exact *p* = 0.004, Fisher’s exact test). Multivariable logistic regression adjusting for age, BMI, diabetes, hypertension, and operative time confirmed this association (Group B vs. A: OR = 0.08, 95% CI = 0.01–0.64, *p* = 0.018; Group C vs. A: OR = 0.08, 95% CI = 0.01–0.64, *p* = 0.018). No significant difference was observed between Group B and Group C (*p* > 0.05, Table 2).

**Table 2:** Number of blood transfusions and the transfusion rate of patients among the three groups.

Groups	Cases	Number of blood transfusions (cases)	Blood transfusion rate (%)
Group A	20	8	40.00
Group B	20	1	5.00
Group C	20	1	5.00
*p*-value			0.004

### Occurrence of complications among the three groups

The overall complication rate was 15.00% (3/20) in Group A, 10% (2/20) in Group B, and 5.00% (1/20) in Group C. No significant difference was observed among groups (*p* = 0.498, Fisher’s exact test). To assess whether this finding was confounded by baseline characteristics, multivariable logistic regression was performed adjusting for age, BMI, history of hypertension, and history of diabetes. After adjustment, the odds of complications remained comparable among the groups. Compared to Group A, the adjusted odds ratio for complications was 0.62 (95% CI: 0.09 to 4.15, *p* = 0.621) for Group B and 0.30 (95% CI: 0.03 to 3.28, *p* = 0.324) for Group C. The adjusted odds ratio for Group C versus Group B was 0.48 (95% CI: 0.04 to 6.12, *p* = 0.572) (see Table 3).

**Table 3:** Occurrence of complications among the three groups.

Groups	Cases	Deep venous thrombosis	Pulmonary embolism	Incision infection	Total occurrence rate
Group A	20	1 (5.00)	1 (5.00)	1 (5.00)	3 (15.00)
Group B	20	0 (0.00)	1 (5.00)	1 (5.00)	2 (10.00)
Group C	20	0 (0.00)	0 (0.00)	1 (5.00)	1 (5.00)
*p*-value					0.498

### Lumbar spine function among the three groups

One year after surgery, the JOA scores of all three groups significantly increased compared to preoperative values (Group A: from 8.6 ± 0.8 to 25.2 ± 2.5, *p* < 0.001; Group B: from 8.6 ± 0.8 to 25.8 ± 2.5, *p* < 0.001; Group C: from 8.6 ± 0.8 to 26.0 ± 2.6, *p* < 0.001). Conversely, the ODI scores of all three groups significantly decreased (Group A: from 41.1 ± 4.1 to 6.5 ± 0.6, *p* < 0.001; Group B: from 41.5 ± 4.1 to 6.4 ± 0.6, *p* < 0.001; Group C: from 41.4 ± 4.2 to 6.3 ± 0.6, *p* < 0.001).

Two-way repeated measures ANOVA revealed no significant group × time interaction for either JOA scores (*F* = 0.42, *p* = 0.658) or ODI scores (*F* = 0.38, *p* = 0.685), indicating that the patterns of improvement over time were similar across all three groups.

To adjust for potential confounders, linear mixed models were performed. After adjusting for age, BMI, history of hypertension, and history of diabetes, the group × time interaction remained non-significant for both JOA scores (*F* = 0.38, *p* = 0.684) and ODI scores (*F* = 0.35, *p* = 0.705), confirming that the similar trajectories of functional recovery among groups were independent of these covariates.

**Figure 3:**
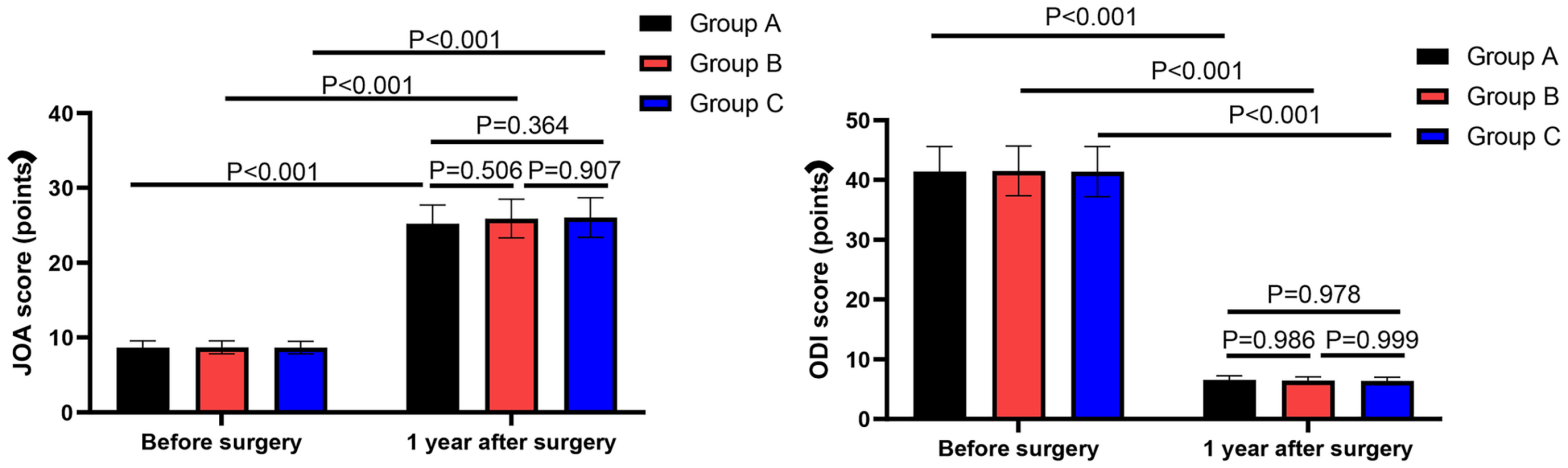
Lumbar spine function among the three groups. Data are shown as mean ± SD. Sample sizes: Group A (*n* = 20), Group B (*n* = 20), Group C (*n* = 20).

However, no significant differences were observed among the three groups in either JOA scores or ODI scores at any time point. These findings are illustrated in Figure 3.

## Discussion

TXA, a lysine derivative and synthetic amino acid (15), exerts competitive inhibitory effects by preventing plasminogen from binding to fibrin, thereby inhibiting fibrin degradation and promoting blood-clot stability to achieve hemostasis (16). As an anti-fibrinolytic drug, it has been widely used in the treatment of post-operative bleeding (17, 18), with various administration methods including oral, topical, and intravenous injection (19).

Several meta-analyses have conducted comparisons regarding the effectiveness and safety profiles of oral versus intravenous administration of TXA in total hip arthroplasty. These studies have consistently demonstrated that oral TXA exhibits comparable blood-conserving effects to intravenous TXA in terms of preventing hemoglobin decline, minimizing total hemoglobin loss, and reducing overall blood loss post-THA. Furthermore, no significant discrepancies were noted between the two administration routes concerning transfusion rates, complication occurrences, or hospital stay durations (20–22). However, the combined efficacy and safety of intravenous and oral TXA administration in TLIF surgery had not been thoroughly elucidated, prompting the initiation of our current investigation.

In our research, we evaluated the effectiveness and safety of three distinct TXA medication strategies in TLIF procedures: preoperative intravenous TXA injection (Group A), preoperative intravenous TXA injection followed by a 3-day course of oral TXA (Group B), and preoperative intravenous TXA injection followed by a 5-day course of oral TXA (Group C). The findings revealed that, in comparison to Group A, both Group B and Group C experienced reduced total blood loss, hidden blood loss, as well as postoperative drainage volumes, along with shorter hospital stays, elevated hemoglobin and hematocrit levels, and decreased blood transfusion rates. Crucially, no significant differences were observed in these measured outcomes between Group B and Group C.

The superior efficacy of the combined intravenous and oral TXA regimens (Group B and Group C) over the single intravenous regimen (Group A) can be attributed to several factors. Firstly, the oral administration of TXA provides a sustained release of the drug into the systemic circulation. After intravenous injection, the drug concentration in the blood decreases rapidly over time due to distribution and metabolism. Oral TXA, when taken after intravenous injection, can maintain a relatively stable drug level in the body, continuously inhibiting fibrinolysis throughout the post-operative period. This continuous inhibition helps to prevent further breakdown of blood clots, thereby reducing both total and hidden blood loss. Secondly, the local environment at the surgical site may also play a role. Although our study did not specifically focus on the local drug concentration, it is reasonable to assume that oral TXA, after absorption into the bloodstream, can reach the surgical site and contribute to the overall anti-fibrinolytic effect. The combined approach may enhance the anti-fibrinolytic activity at the site of injury, where fibrinolysis is more likely to occur during the healing process.

The lack of significant differences between Group B and Group C in terms of the main observation indicators suggests that a 3-day oral administration of TXA after intravenous injection may be sufficient to achieve the optimal anti-fibrinolytic effect in TLIF surgery. Extending the oral administration to 5 days did not lead to further significant improvements. This could be because the fibrinolytic activity in the post-operative period of TLIF surgery is mainly concentrated in the early days after the operation. After 3 days, the body’s natural healing processes and the initial anti-fibrinolytic effects of TXA may have already stabilized the blood-clot environment, and additional oral TXA does not provide further benefits.

Consistent with our findings, Cankaya et al. (23) conducted a comparison between the combined application (oral plus topical) of TXA and the sole topical use of TXA in total knee arthroplasty. Their research revealed that the combined regimen of oral and topical TXA is not only safe and effective but also cost-efficient in minimizing blood loss and lowering transfusion rates following total knee arthroplasty. This further corroborates the notion that a multi-modal strategy for TXA administration can amplify its anti-fibrinolytic properties.

Regarding safety outcomes, no statistically significant differences were observed among the three groups in terms of intraoperative blood loss, drainage tube removal time, coagulation function indicators, fibrinolytic indicators, or complication rates (including DVT, PE, and infection). Similarly, long-term functional outcomes (JOA and ODI scores) at 1-year post-surgery showed no significant differences among groups.

However, these findings must be interpreted with caution. With only 20 patients per group (60 total), our study is substantially underpowered to detect differences in rare but clinically important adverse events such as DVT, PE, or surgical site infections. The absence of statistically significant differences does not constitute evidence of equivalence or safety among the three regimens. Given the low event rates expected for these complications, a much larger sample size would be required to draw meaningful conclusions about comparative safety.

The comparable results in coagulation parameters (PT, APTT, D-dimer, and FIB) suggest that the different TXA regimens did not produce detectable systemic coagulation abnormalities in this small cohort, but this does not rule out the possibility of rare thrombotic events. Similarly, the lack of differences in intraoperative blood loss likely reflects that the preoperative intravenous TXA administered to all three groups provided adequate anti-fibrinolytic protection during the surgical procedure itself, rather than indicating equivalence of the postoperative oral regimens.

Therefore, while our findings are reassuring in that no safety signals were detected, we cannot conclude that the three regimens are “equally safe.” Larger, adequately powered randomized controlled trials are essential to properly assess the comparative safety profiles of different TXA administration strategies in TLIF surgery.

Our study has several important limitations. Firstly, the prospective observational cohort design (non-randomized) increases the risk of confounding, despite multivariable adjustment for key variables (age, BMI, diabetes, hypertension, operative time). Randomized controlled trials (RCTs) are needed to confirm causal relationships. Secondly, the small sample size (*n* = 60) limits statistical power for safety outcomes and subgroup analyses. Thirdly, the study is single-center, which may limit generalizability to other institutions or populations. Fourthly, no blinding was performed (patients, surgeons, outcome assessors), introducing potential performance and detection bias. Fifthly, the study is underpowered to assess rare adverse events, and safety conclusions are tentative. Finally, we only evaluated two postoperative oral durations (3 and 5 days); future studies could explore different doses or frequencies to optimize TXA use.

## Conclusion

This single-center prospective observational cohort study, with multivariable adjustment for confounding factors, provides preliminary evidence that intravenous TXA followed by 3 days of oral TXA may reduce total blood loss, hidden blood loss, postoperative drainage volume, hospital stay, and transfusion rates in patients undergoing TLIF surgery, compared to intravenous TXA alone. However, these findings should be interpreted with caution due to the observational design, small sample size (*n* = 60), and lack of blinding.

Regarding safety, the study is substantially underpowered to assess rare but clinically important adverse events such as DVT, PE, or infection. The absence of statistically significant differences in complication rates does not imply equivalence or safety among the three regimens. Larger, adequately powered multi-center randomized controlled trials are needed to both validate the efficacy findings and properly evaluate the comparative safety profiles of combined intravenous and oral TXA strategies.

Pending confirmation from higher-quality evidence, the current results support the rational use of combined intravenous and 3-day oral TXA as a potential cost-effective strategy for perioperative blood management in TLIF surgery, with the understanding that safety conclusions remain tentative.

## Data Availability

The original contributions presented in the study are included in the article/Supplementary material, further inquiries can be directed to the corresponding author.

## Supplementary material

FIGURE S1 CONSORT flow diagram.

## References

[1] Ravindra VM Senglaub SS Rattani A Dewan MC Härtl R Bisson E . Degenerative lumbar spine disease: estimating global incidence and worldwide volume. Global Spine J. (2018) 8:784–94. doi: 10.1177/2192568218770769, 30560029 PMC6293435

[2] Chen X Zheng Z Lin J. Clinical effectiveness of conservative treatments on lumbar spinal stenosis: a network meta-analysis. Front Pharmacol. (2022) 13:859296. doi: 10.3389/fphar.2022.859296, 35734403 PMC9207476

[3] Chang MC Kim GU Choo YJ Lee GW. Transforaminal lumbar interbody fusion (TLIF) versus oblique lumbar interbody fusion (OLIF) in interbody fusion technique for degenerative spondylolisthesis: a systematic review and meta-analysis. Life. (2021) 11:696. doi: 10.3390/life11070696, 34357068 PMC8305484

[4] Rathbone J Rackham M Nielsen D Lee SM Hing W Riar S . A systematic review of anterior lumbar interbody fusion (ALIF) versus posterior lumbar interbody fusion (PLIF), transforaminal lumbar interbody fusion (TLIF), posterolateral lumbar fusion (PLF). Eur Spine J. (2023) 32:1911–26. doi: 10.1007/s00586-023-07567-x, 37071155

[5] Lin S Liu S Li Y Yan Y Ye H. Hidden blood loss of minimally invasive transforaminal lumbar interbody fusion of lumbar degenerative diseases in patients with osteoporosis: a retrospective study. J Orthop Surg Res. (2024) 19:798. doi: 10.1186/s13018-024-05297-4, 39593090 PMC11600765

[6] Ding Y Chen H Wu G Xie T Zhu L Wang X. Comparison of efficacy and safety between unilateral biportal endoscopic transforaminal lumbar interbody fusion versus uniportal endoscopic transforaminal lumbar interbody fusion for the treatment of lumbar degenerative diseases: a systematic review and meta-analysis. BMC Musculoskelet Disord. (2024) 25:1037. doi: 10.1186/s12891-024-08146-x, 39702176 PMC11660777

[7] Xie C Zhang L Cai G Su Y Wang P Luo H. Efficacy and safety of topical versus intravenous tranexamic acid in spinal surgery: a systematic review and meta-analysis. BMC Surg. (2025) 25:15. doi: 10.1186/s12893-024-02743-2, 39789531 PMC11714873

[8] Xie C Ren Y Chen X Zhu Y Jiang J Lu B . The efficacy and safety of topical combined with intravenous administration of tranexamic acid in spine surgery: a systematic review and meta-analysis. BMC Musculoskelet Disord. (2024) 25:1074. doi: 10.1186/s12891-024-08191-6, 39725950 PMC11673842

[9] Zheng B Li G Li C Zhu Z Liu H. Comparing the efficacy and safety of oral versus intravenous tranexamic acid in spine surgery: a systematic review and meta-analysis of randomized controlled trials. Neurosurg Rev. (2025) 48:470. doi: 10.1007/s10143-025-03637-4, 40448910

[10] Choi H Kim DW Jung E Kye YC Lee J Jo S . Impact of intravesical administration of tranexamic acid on gross hematuria in the emergency department: a before-and-after study. Am J Emerg Med. (2023) 68:68–72. doi: 10.1016/j.ajem.2023.03.020, 36948083

[11] Morris JL Letson HL McEwen P Biros E Dlaska C Hazratwala K . Comparison of intra-articular administration of adenosine, lidocaine and magnesium solution and tranexamic acid for alleviating postoperative inflammation and joint fibrosis in an experimental model of knee arthroplasty. J Orthop Surg Res. (2021) 16:726. doi: 10.1186/s13018-021-02871-y, 34930351 PMC8686251

[12] King L Randle R Dare W Bernaitis N. Comparison of oral vs. combined topical/intravenous/oral tranexamic acid in the prevention of blood loss in total knee arthroplasty: a randomised clinical trial. Orthop Traumatol Surg Res. (2019) 105:1073–7. doi: 10.1016/j.otsr.2019.06.008, 31473130

[13] Waddell BS Zahoor T Meyer M Ochsner L Chimento G. Topical tranexamic acid use in knee periprosthetic joint infection is safe and effective. J Knee Surg. (2016) 29:423–9. doi: 10.1055/s-0035-1564599, 26408993

[14] He B Li Y Xu S Ou Y Zhao J. Tranexamic acid for blood loss after transforaminal posterior lumbar interbody fusion surgery: a double-blind, placebo-controlled, randomized study. Biomed Res Int. (2020) 2020:8516504. doi: 10.1155/2020/8516504, 32855972 PMC7443232

[15] Kumar M Venishetty S Jindal A Bihari C Maiwall R Vijayaraghavan R . Tranexamic acid in upper gastrointestinal bleed in patients with cirrhosis: a randomized controlled trial. Hepatology. (2024) 80:376–88. doi: 10.1097/HEP.0000000000000817, 38441903

[16] Issa SM Sang Y Granko HA Wolberg AS. High tranexamic acid concentrations alter fibrin network structure and biophysical properties. J Thromb Haemost. (2025) 23:3001–6. doi: 10.1016/j.jtha.2025.05.012, 40412719 PMC12571112

[17] Ker K Sentilhes L Shakur-Still H Madar H Deneux-Tharaux C Saade G . Tranexamic acid for postpartum bleeding: a systematic review and individual patient data meta-analysis of randomised controlled trials. Lancet. (2024) 404:1657–67. doi: 10.1016/S0140-6736(24)02102-0, 39461793 PMC12197804

[18] Cheema HA Ahmad AB Ehsan M Shahid A Ayyan M Azeem S . Tranexamic acid for the prevention of blood loss after cesarean section: an updated systematic review and meta-analysis of randomized controlled trials. Am J Obstet Gynecol MFM. (2023) 5:101049. doi: 10.1016/j.ajogmf.2023.101049, 37311484

[19] Park LJ Marcucci M Ofori SN Borges FK Nenshi R Kanstrup CTB . Safety and efficacy of tranexamic acid in general surgery. JAMA Surg. (2025) 160:267–74. doi: 10.1001/jamasurg.2024.6048, 39813061 PMC11904705

[20] Zhang LK Ma JX Kuang MJ Zhao J Wang Y Lu B . Comparison of oral versus intravenous application of tranexamic acid in total knee and hip arthroplasty: a systematic review and meta-analysis. Int J Surg. (2017) 45:77–84. doi: 10.1016/j.ijsu.2017.07.097, 28755884

[21] Daghmouri MA Salmeron F Lahdheri AA Da Costa AC Chaouch MA. Efficacy and safety of oral versus intravenous administration of tranexamic acid in elective total hip arthroplasty: a systematic review and meta-analyses of randomized controlled trials. J Arthroplasty. (2026) 41:265–273.e1. doi: 10.1016/j.arth.2025.04.042, 40306560

[22] Sun C Zhang X Chen L Deng J Ma Q Cai X . Comparison of oral versus intravenous tranexamic acid in total knee and hip arthroplasty: a GRADE analysis and meta-analysis. Medicine (Baltimore). (2020) 99:e22999. doi: 10.1097/MD.0000000000022999, 33126380 PMC7598783

[23] Cankaya D Dasar U Satilmis AB Basaran SH Akkaya M Bozkurt M. The combined use of oral and topical tranexamic acid is a safe, efficient and low-cost method in reducing blood loss and transfusion rates in total knee arthroplasty. J Orthop Surg (Hong Kong). (2017) 25:2309499016684725. doi: 10.1177/2309499016684725, 28176599

