# Supplementary figures and images for: Clinical application of different administration methods of tranexamic acid in transforaminal lumbar interbody fusion surgery
Source: Front Med (Lausanne). 2026 Mar 30;13:1777031. doi: 10.3389/fmed.2026.1777031 (PMC13070914; doi:10.3389/fmed.2026.1777031)

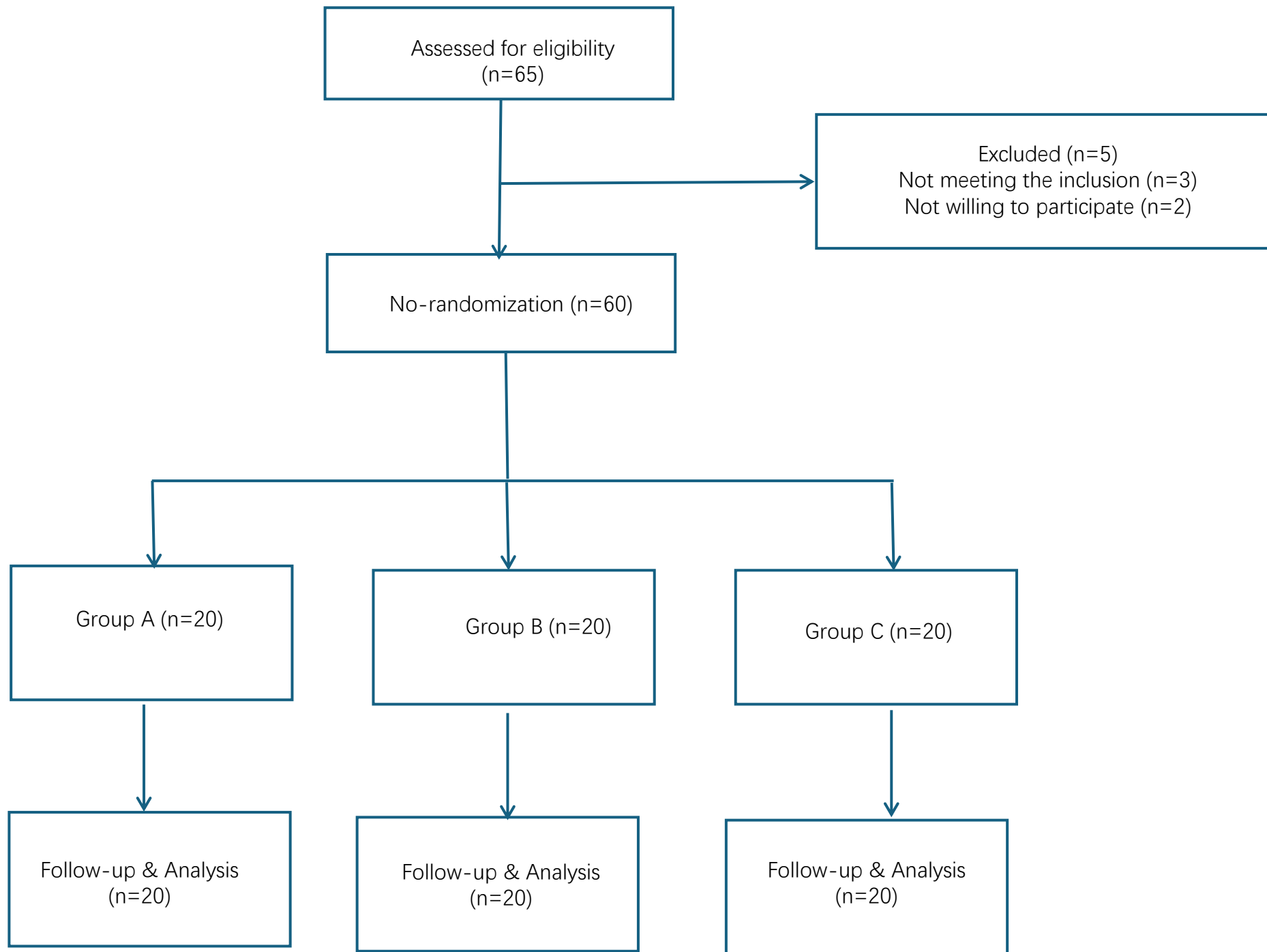

Supplement: FIGURE S1 — CONSORT flow diagram. [file Data_Sheet_1.pdf]
